# Ex Utero Intrapartum Treatment for Fetal Oropharyngeal Cyst

**DOI:** 10.1155/2010/273410

**Published:** 2010-01-20

**Authors:** Allen W. Ayres, Suzanne K. Pugh

**Affiliations:** Division of Maternal-Fetal Medicine, Department of Obstetrics and Gynecology, Naval Medical Center Portsmouth, 620 John Paul Jones Circle, Portsmouth, VA 23708, USA

## Abstract

*Background*. A prenatally diagnosed fetal anomaly that could compromise the fetal airway at delivery can be managed safely with the ex utero intrapartum treatment (EXIT) procedure. *Case*. A 26-year-old healthy primigravida was diagnosed during her midtrimester anatomic ultrasound survey with a fetal oropharyngeal cystic structure located at the base of the tongue. The neonatal airway was successfully secured intrapartum using the EXIT procedure. *Conclusion*. Maintenance of fetoplacental circulation until the fetal airway is secured has been described for a multitude of fetal anomalies including cystic hygroma and teratoma. The literature also recounts its use for the reversal of tracheal plugging for congenital diaphragmatic hernia. A multidisciplinary approach to the antenatal and intrapartum care is essential for the successful management of these cases.

## 1. Introduction

A cystic mass at the base of the fetal tongue diagnosed antenatally is uncommon, relegated to case reports in the literature. Dr. Hartnick et al. [1] recently published a case report of a pregnancy complicated with a cystic mass in the mouth of the fetus measuring 2.0 cm by 2.3 cm noted on ultrasound, and because of the concern for the neonatal airway, the patient was delivered by an EXIT procedure. The procedure was successful with good outcomes for both the mother and the neonate. This case also demonstrated the importance of managing this case using a multidisciplinary approach [1].

Fetal airway obstruction at delivery is a potentially fatal complication. If a fetal oropharyngeal anomaly is noted antepartum, the neonatal airway can be secured intrapartum using the EXIT procedure, thus reducing potential neonatal morbidity and mortality [2]. Mychaliska and colleagues first described the systematic ex utero intrapartum treatment procedure to secure a fetal airway in 1997 [3]. Optimizing uteroplacental blood flow with the use of inhalational anesthetic agents and uterine tocolytics allows for the maintenance of fetal oxygenation.

We report a case of a pregnancy complicated by a fetal oropharyngeal cyst and successfully securing the fetal airway using the EXIT procedure. This report demonstrates the effectiveness of this procedure in securing the fetal airway while maintaining the fetoplacental circulation and adequate fetal oxygenation. 

At our institution, an IRB approval is not required for a case report.

## 2. Case

**Figure 1:**
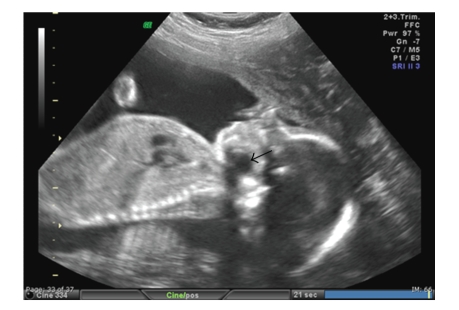
Ultrasound image at 26-week gestation demonstrating oropharyngeal cyst (black arrow).

A 26-year-old primigravida presented to the Maternal Fetal Medicine (MFM) clinic for consultation regarding a 1 cm cystic structure located at the floor of the fetal mouth. The structure had been visualized on an ultrasound in the radiology department at 21-week gestation. The patient was healthy without any significant medical or family history. Her prenatal laboratory evaluation was normal, and her medications included prenatal vitamins. An ultrasound, performed by the MFM department, at 25-week gestation, revealed a 1.4 cm × 1.4 cm cystic structure located posterior and inferior to the tongue (Figure 1). The lips, nose, mandible, palate, and profile were all normal. The differential diagnosis included a thyroglossal duct cyst or a branchial duct cyst. Magnetic resonance imaging at 26-week gestation revealed a 1.4 cm × 2 cm × 1.9 cm midline cystic lesion located at the base of the tongue/floor of the mouth. The differential diagnosis was then expanded to include epidermoid cyst and lymphangioma.

The case was discussed in the weekly NICU/MFM conference with the pediatric ENT physician in attendance. The options of securing the airway during an EXIT procedure or post delivery after clamping the umbilical cord were discussed. Since the oropharyngeal cyst had increased to 3.1 cm, the pediatric otolaryngologist felt that the fetal airway would be more safely managed during an EXIT procedure, allowing more time for controlled bronchoscope guided intubation, and if necessary, tracheotomy, while the fetus is being perfused and oxygenated with an intact fetal-placental unit. The team was kept informed of the progress during the antenatal course and would be available at all times for the delivery. In addition, the pediatric otolaryngologist consulted the pediatric anesthesiologist who would also be present during the EXIT procedure. Furthermore, the pediatric cardiologist would be in attendance for the delivery to monitor the fetal cardiac function. The EXIT procedure was scheduled for 39 weeks of gestation. Serial MFM ultrasounds were obtained at 32 and 36 weeks of gestation revealing dimensions of the mass at 2.1 cm × 1.1 cm × 2.1 cm and 1.9 cm × 2.3 cm × 3.1 cm, respectively. The estimated fetal weight at the last ultrasound was 2584 grams, 10th–50th percentile (Alexander growth curve) with minimal polyhydramnios (single deepest pocket, 8.34 cm). The placenta was posterior in location. 

The patient presented in active labor at 37 + 3 weeks of gestation; the cervix was 6 cm dilated and 80% effaced. The EXIT team members were notified emergently. Fetal well being was reassured by a reactive nonstress test (NST). Intravenous magnesium sulfate was administered for tocolysis while the team was assembled. The anesthesiologist placed an arterial line for hemodynamic monitoring intraoperatively. Preoperative hemoglobin was 13.3 g/dL. A type and cross for four units of packed red blood cells were obtained, and the blood products were brought to the operating room. The pediatric anesthesiologist and the otolarnygologist recommended the use of a fetal paralytic agent prior to delivery to prevent the fetus from gasping at the time of delivery that would make the intubation process potentially more complicated and risky. Under ultrasound guidance, rocuronium was injected intramuscularly into the fetal thigh for paralysis. Ultrasound examination was continued until fetal movement ceased, occurring at approximately four minutes post injection.

**Figure 2:**
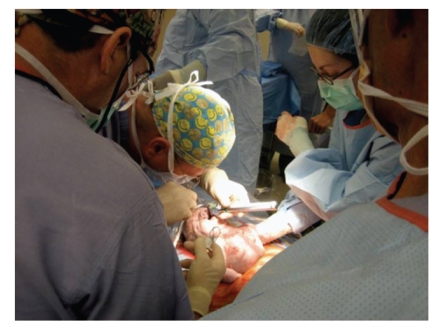
Intraoperative image of EXIT procedure. The fetus is delivered to level of upper abdomen with the fetal head and neck stabilized by the obstetrician. The pediatric anesthesiologist performs direct laryngoscopy.

The patient was then taken to the operating room where general endotracheal anesthesia was administered via rapid sequence induction with thiopental and succinylcholine. Paralysis was subsequently maintained with vecuronium. Deep inhalation anesthesia was achieved with high-dose isoflurane. Maternal laparotomy was then performed via a low transverse abdominal incision to expose the uterus. A low transverse incision was made in the lower uterine segment, and the infant was delivered to the level of the upper abdomen as shown in Figure 2. Continuous fetal cardiac monitoring was achieved with fetal transthoracic echocardiogram, monitored by the pediatric cardiologist in the operating room. Initially, two attempts at intubation with the laryngoscope were made but were unsuccessful. The vocal cords could not be visualized to pass the endotracheal tube. The third attempt was done via bronchoscope guidance and was successful. The mass was posterior in the oropharynx obstructing the view of the vocal cords. The pediatric otolaryngologist performed the bronchoscopy guided placement of the endotracheal tube. Placement of the endotracheal tube was confirmed and the tube was secured. The remainder of the infant's body was then delivered, the umbilical cord was divided, and the infant was taken to the warmer for attendance by the neonatology team. The total elapsed time of the EXIT procedure was 18 minutes. 

The halogenated anesthetic was discontinued and oxytocin infusion was initiated following delivery of the placenta. Uterine massage and 40 units of intravenous oxytocin effected adequate uterine tone. The hysterotomy incision was repaired in two layers. Estimated blood loss from the procedure was 1200 mL. No intraoperative fetal or maternal complications occurred. The mother's postoperative recovery was uncomplicated. She was discharged on the second postoperative day and had a normal 6-week postpartum checkup.

A neonatal MRI confirmed the cystic mass, arising from the midline at the base of the tongue, measuring 2.4 cm × 2.2 cm × 1.6 cm. The appearance of the mass was most consistent with a thyroglossal duct cyst. The cystic mass was initially drained. But, one week later, it recurred and then was surgically excised. Pathology was consistent with an embryologic remnant cyst. Follow-up examinations revealed a healthy, normally developing infant.

## 3. Comments

The EXIT procedure was initially designed to reverse the tracheal occlusion that was done antenatally in a fetus with severe congenital diaphragmatic hernia [3]. Because it is able to provide a stable fetal hemodynamic environment for a prolonged period, the EXIT procedure has been applied to the treatment of a variety of fetal conditions at delivery [4]. 

In order to achieve successful fetal oxygenation and the deep maternal anesthesia that provides fetal anesthesia during the EXIT procedure, it is necessary to maintain uterine hypotonia. In this case, uterine hypotonia was achieved using isoflurane intraoperatively. Additional methods include intravenous terbutaline or nitroglycerin [3]. 

To ensure fetal paralysis during the EXIT procedure (as recommended by the pediatric anesthesiologist), we administered intramuscular rocuronium into the fetal thigh under ultrasound guidance preoperatively. Fetal paralysis occurred in four minutes after the injection and was confirmed by ultrasound. To our knowledge, this is the first report using a fetal paralytic agent for an EXIT procedure. The decision was made based on the above reason—the clinical opinion of the pediatric subspecialists. 

The EXIT procedure is associated with maternal risks that are well documented in the literature. Since it is necessary to maintain uterine hypotonia to maximize uteroplacental blood flow, the mother is at risk for hemorrhage and placental abruption.

The surgeons and anesthesia providers must have a low threshold for terminating the EXIT procedure before a significant loss of blood occurs. 

In one large case series of thirty-one patients, the average time on placental bypass was 30.7 minutes, with a range from 8 to 66 minutes. The average blood loss was 848 mL [3]. In our case, securing the airway required eighteen minutes, and our estimated blood loss was 1200 mL. 

It is important to deliver the fetus only to a level where the airway and neck can be accessed for evaluation. When the remainder of the fetal body fills the intrauterine cavity, the possibility of placental detachment is decreased. Other authors have described a technique similar to amnioinfusion to allow for persistent uterine distention with a lower risk of placental separation [4].

The importance of a multispecialty approach with constant communication cannot be overemphasized. In our case, despite a procedure which was scheduled to occur at 39 weeks of gestation, our patient presented in active labor at 37 weeks. The multiple team members had to assemble promptly to perform the EXIT procedure. This multispecialty team had met on several occasions during the course of this pregnancy, and thus, the role of each subspecialist was clear. This ensured a smooth assembly of all necessary personnel and equipment on short notice.

In summary, in this case of a fetal oropharyngeal mass, the fetal airway was successfully and safely secured using the EXIT procedure. In addition, this case demonstrated the importance of a multidisciplinary approach in managing such cases.
